# Enhanced HIV immune responses elicited by an apoptotic single-cycle SHIV lentivector DNA vaccine

**DOI:** 10.3389/fcimb.2025.1481427

**Published:** 2025-04-10

**Authors:** Deepanwita Bose, Kenneth A. Rogers, Lisa M. Shirreff, Yahia Chebloune, Francois J. Villinger

**Affiliations:** ^1^ New Iberia Research Center, University of Louisiana at Lafayette, New Iberia, LA, United States; ^2^ Laboratoire Pathogénèse et Vaccination Lentivirales (PAVAL) Lab., Institut National de Recherche d’Agriculture et Environnement, Université Grenoble Alpes, Saint Martin d’Hères, France

**Keywords:** SHIV, DNA vaccine, lentivector, apoptotic gene, immune responses, mouse immunization

## Abstract

**Background:**

HIV remains a major public health issue in spite of antiretroviral therapy (ART). An innovative vaccine that can induce long-lasting and effective immunity is required to curb the persistently high numbers of new infections worldwide.

**Methods:**

A novel DNA vaccine was generated using a Simian-Human Immunodeficiency Virus (SHIV) backbone with a Zambian T/F clade C envelope and under the control of the caprine arthritis encephalitis virus long terminal repeats (LTRs) for constitutive expression. Due to the deleted integrase, this DNA vaccine “CSH-DIN-T/F Z331” performs only a single replication cycle. To increase immunogenicity, the co-expression of apoptotic genes (BAX, BAK, or caspase 8) incorporated at the end of Pol was tested to promote the release of apoptotic bodies taken up by dendritic cells leading to cross-presentation of antigen. The three vaccines (CSH-DIN-T/F Z331-BAX, CSH-DIN-T/F Z331-BAK, and CSH-DIN-T/F Z331-Cas8) were tested *in vitro* for expression and *in vivo* in BALB/cJ mice for immunogenicity.

**Results:**

Transduced HEK293 cells co-cultured with CEMx174 confirmed the single replication cycle of the DNA vaccine and the induction of apoptosis by CSH-DIN-T/F Z331-Cas8 based on Annexin V expression. BALB/cJ mice were immunized with a combined intramuscular + intradermal/electroporation approach. Intracellular cytokine staining (ICS) from splenocytes collected 12 weeks post-prime/6 weeks post-boost demonstrated a clear superiority of caspase 8 expressing construct over the others, with higher proportions of IFN-γ-, IL-2-, and IL-21-producing CD8 T cells specific to Env, Gag, and Nef. The kinetics of immune response after various immunization schedules were also investigated.

**Conclusion:**

This novel single-cycle DNA vaccine with apoptotic genes demonstrated an enhanced immunogenicity primarily for antigen-specific CD8+ T-cell responses.

## 1 Introduction

According to United Nations Programme on HIV and AIDS (UNAIDS) statistics, by the end of 2023, 39.9 million people were living with HIV globally, with 1.3 million new infections and 9.2 million people still without access to antiretroviral therapy (ART) (https://www.unaids.org/en/resources/fact-sheet). Daily ART has been highly effective in controlling virus replication and bringing virus replication down to undetectable and untransmittable (U=U) levels. However, ART has failed to fully eliminate HIV due to the rapid establishment of persistent reservoirs in lymphoid organs and anatomical sanctuaries such as the brain, testis, and gut-associated lymphoid tissues (Castro-Gonzalez et al., 2018; Whyte-Allman and Bendayan, 2020).

An ideal HIV vaccine would protect people from acquiring infection and control the virus post-infection in people living with HIV. There have been 12 HIV vaccine and passive antibody efficacy clinical trials over 20 years, with only two having had positive outcomes. The 2009 Thai RV144 Prime-Boost, ALVAC-AIDSVAX, B/E trial had a modest 31.2% efficacy (Vaccari et al., 2010), and the 2021 Antibody Mediated Prevention (AMP) studies using the VRCO1 monoclonal antibody reduced the risk of acquisition for patients exposed to HIV strains highly sensitive to VRCO1 neutralization but not to the majority of strains (Corey et al., 2021).

It is essential to develop innovative strategies for vaccines against HIV. In this regard, a vaccination strategy consisting of DNA is safe, simple, stable, and scalable and has minimal storage and transport requirements. However, immunization with HIV DNA vaccines, while effective in rodents, has induced only marginal responses in humans or large animal models. However, these responses are improved when DNA immunization is combined with electroporation to promote cell transfection (Sarwar et al., 2015; Hannaman et al., 2016; Todorova et al., 2017; Elizaga et al., 2018; Tebas et al., 2019; Hooper et al., 2020; Sefidi-Heris et al., 2020; Ahn et al., 2022; Houser et al., 2022; Kraynyak et al., 2022). We developed a SHIV DNA vaccine based on modified lentiviral genomes to better mimic the early stages of natural exposure to HIV and the production of viral proteins.

Previously, a unique SHIV DNA vaccine CAL-SHIV-IN^−^ was developed with i) deleted integrase limiting replication to a single cycle in target cells without integration (Arrode-Bruses et al., 2014; Moussa et al., 2015). This single cycle leads to the production of viral antigens that assemble into virus-like particles (VLPs) and pseudo-infectious particles (Arrode-Bruses et al., 2014; Moussa et al., 2015). ii) The vaccine is under the control of caprine arthritis encephalitis virus (CAEV) long terminal repeats (LTRs), which deliver a constitutive expression of viral proteins. CAEV LTRs are Tat independent and are reviewed in more detail in the review (Bose et al., 2015). *In vivo* studies with a previous version of this DNA vaccine using combined intramuscular (i.m.) and intradermal (i.d.) injections plus electroporation have demonstrated the enhancement of DNA uptake up to 10- to 100-fold into cells while also causing low-level inflammation (Diehl et al., 2013; Kisakov et al., 2024). This DNA vaccine led to durable central and effector SHIV-specific memory T-cell responses elicited after a single immunization, which endured up to 80 weeks in the absence of a booster (Arrode-Bruses et al., 2014). All vaccinated cynomolgus macaques rapidly controlled viral replication up to 1 year after the challenge. Remarkably, this challenge control correlated with vaccine-specific T-cell precursors with high proliferation capacity (PHPC) in all vaccinated macaques (Arrode-Bruses et al., 2014). The addition of IL-7 or IL-15 in the construct brought marginal improvement in the immune responses (Leroy et al., 2022). This vaccine nevertheless left room for improvement, in particular, if a more virulent model (e.g., rhesus macaques infected with novel SHIV isolates) was to be used. Thus, to further enhance cytotoxic T-cell immunity, we hypothesized that adding an apoptotic gene in the vaccine may trigger rapid apoptosis of transduced cells, leading to the uptake of apoptotic bodies by dendritic cells, thereby increasing antigen presentation as well as cross-presentation of antigen (Sasaki et al., 2002; Scheffer et al., 2003; Kim et al., 2004; Parsania et al., 2010; Mekonnen et al., 2020). Apoptosis is mediated by either the extrinsic cell death pathway (via caspase 8) or the mitochondrial/intrinsic pathway (via BCL2 family member BAK/BAX).

For this study, we developed a novel DNA vaccine using a SHIV backbone with a transmitted founder (T/F) Z331 clade C envelope from a Zambian cohort (GenBank KR820323) (Deymier et al., 2015). T/F viruses were selected through the genetic bottleneck during transmission, as they are CCR5 tropic, exhibit less Env glycosylation, and preferentially bind α4β7 (Parrish et al., 2013; Deymier et al., 2015). The novel DNA vaccine under the control of CAEV LTRs (CSH-DIN-T/F Z331) ensures the constitutive expression of viral proteins during its single-cycle replication with no integration due to the deletion of the integrase gene. Additionally, the apoptotic genes BAX, BAK, and caspase 8 were included with a T2A at the end of Pol. The three vaccines—CSH-DIN-T/F Z331-BAX, CSH-DIN-T/F Z331-BAK, and CSH-DIN-T/F Z331-Cas8—were tested *in vitro* for expression, and CSH-DIN-T/F Z331-BAX and CSH-DIN-T/F Z331-Cas8 were tested *in vivo* in BALB/cJ mice for immunogenicity.

## 2 Materials and methods

### 2.1 DNA vaccine construction

**Figure 1:**
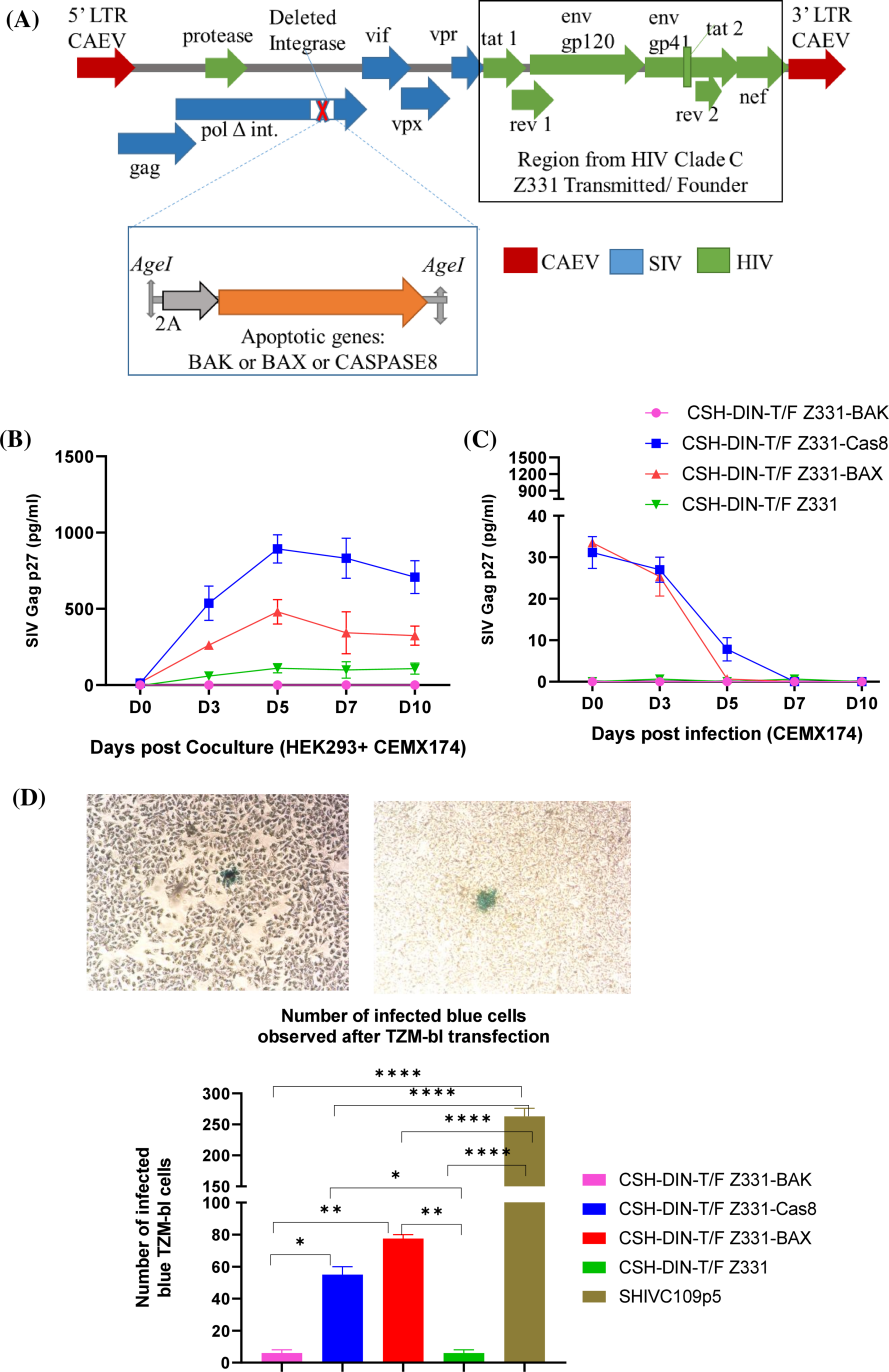
Development and *in vitro* characterization of lenti DNA vaccines. **(A)** Schematics of lenti DNA vaccine CSH-DIN-T/F Z331 with the backbone of SHIV, CAEV LTRs, and deleted integrase; the region from *tat*–*nef* is derived from T/F Z331 clade C HIV. Using the unique restriction site *Age*I, the cassette including apoptotic genes with upstream T2A is inserted to develop CSH-DIN-T/F Z331-BAX, CSH-DIN-T/F Z331-BAK, or CSH-DIN-T/F Z331-Cas8. **(B)** For all the lenti DNA vaccine prototypes, SIV Gag p27 was determined from the supernatants of the transiently transfected HEK293 co-cultured with CEMx174 cells in two independent experiments **(C)** and with the supernatants of CEMx174 infected with day 2 HEK293 transiently transfected cells. **(D)** Infectivity of all the lenti vector DNA vaccines was determined by transiently transfecting the TZM-bl cells and staining after 72 hrs to quantitate the blue infected cells. Representative image of blue infected cell from CSH-DIN-T/F Z331 is in the top panel. The number of infected blue cells from two independent experiments is summarized in the graph. SHIV-C109p5, a replication-competent virus, is a positive control. Results were compared using Tukey’s multiple comparison test and one-way ANOVA. Error bars represent the standard error of mean (s.e.m). *p < 0.05, **p < 0.01, ****p < 0.0001. CAEV, caprine arthritis encephalitis virus; LTRs, long terminal repeats.

The CSH-DIN-T/F Z331 DNA vaccine construction details were previously published (Bose, 2017). Briefly, the CSH-DIN-T/F Z331 DNA vaccine was generated using the SHIV backbone under the control of CAEV LTRs and deleted integrase gene (Arrode-Bruses et al., 2014) with the substitution of the *tat* to *nef* region containing the *env* gene of the T/F clade C envelope (Deymier et al., 2014). Similar to the parent CAL-SHIV-IN^−^, this DNA vaccine can only achieve a single-cycle replication without integration (**Figure 1A**).

The three vaccines CSH-DIN-T/F Z331-BAX, CSH-DIN-T/F Z331-BAK, and CSH-DIN-T/F Z331-Cas8 were constructed by gene synthesis by inserting a T2A coding sequence driving the translation of BAX, BAK, or caspase 8 at the end of the reverse transcriptase ORF using the *Age*I restriction site (GenBank references for BAX, BAK, and caspase 8 are XM_015124399.2, XM_015136057.2, and NM_001284129.1, respectively) (Thermo Fisher Scientific, Waltham, MA, USA; GENEART).

The synthesized gene inserts were digested with *Age*I and ligated to the vector CSH-DIN-T/F Z331. The ligated products were transformed in One Shot Stbl3 chemically competent *Escherichia coli* (Invitrogen, Carlsbad, CA, USA; #C737303). The colonies were screened and amplified using midi-preps, and the inserted region was Sanger sequenced (Eurofins Genomics, Louisville, KY, USA). Each plasmid sequence was verified further via whole plasmid sequencing using Next-Generation Sequencing (NSG) generation 3 technology by Eurofins Genomics.

### 2.2 Cell culture

Cell lines (HEK293, TZM-bl, and CEMx174) were obtained from National Institutes of Health (NIH) (AIDS Reagent Program, NIAID, Bethesda, MD, USA), and cells were cultured according to the recommended culture conditions.

#### 2.2.1 HEK293 transfection, co-culture, and infection of CEMx174

In a 6-well plate, 5 × 10^5^ adherent HEK293 cells were transiently transfected using 9 µg polyethylenimine (PEI) and 3 µg DNA (CSH-DIN-T/F Z331-BAX, CSH-DIN-T/F Z331-BAK, CSH-DIN-T/F Z331-Cas8, or CSH-DIN-T/F Z331) and incubated in tissue culture conditions for 8 hrs. The transfection medium was removed, and a fresh medium was added. Forty-eight hours post-transfection, CEMx174 cells were added to co-culture with the HEK293 cells. For infection, 100 µL of 48-hrs post-transfection supernatant was used to inoculate CEMx174. Days 3, 5, 7, and 10 supernatants were collected from the co-cultures and infections for SIV Gag p27 protein quantification using ELISA (ABL, Inc., Rockville, MD, USA; #SKU 5436). ELISA was conducted according to the manufacturer’s instructions followed by reading the absorbance at 450 nm using a BioTek (Winooski, VT, USA) Synergy HT microplate reader.

#### 2.2.2 TZM-bl transfection/infected and quantifying number of infected cells

The TZM-bl indicator cell line was used to quantitate the viral construct using β-gal as a reporter. In a 6-well plate, 5 × 10^5^ adherent TZM-bl cells were transiently transfected using 9 µg PEI and 3 µg DNA (CSH-DIN-T/F Z331-BAX, CSH-DIN-T/F Z331-BAK, CSH-DIN-T/F Z331-Cas8, or CSH-DIN-T/F Z331). In parallel, an infectious virus SHIV-C109p5 was used to infect the TZM-bl cells as the positive control (Bose et al., 2024). The transfection medium was removed, and fresh medium was added after 8 hrs. Seventy-two hours post-transfection, the cells were washed twice with Phosphate-buffered saline (PBS) and fixed with 4% Paraformaldehyde (PFA). Cells were rinsed once and incubated in PBS containing 5 mM MgCl_2_. Cells were stained with 1 mg/mL X-Gal in a staining solution containing 5 mM K_3_Fe(CN)_6_ and 2 mM K_4_Fe(CN)_6_. The cells were incubated for 1 hr at 37°C and then fixed again in 4% PFA for 15 min before washing in PBS and drying. The number of blue cells was counted and reported.

#### 2.2.3 Evaluating transcription and translation of apoptotic genes

Half a million HEK293 cells were transiently transfected using 9 µg PEI and 3 µg DNA (CSH-DIN-T/F Z331-BAX, CSH-DIN-T/F Z331-BAK, CSH-DIN-T/F Z331-Cas8, or CSH-DIN-T/F Z331). Forty-eight hours post-transfection, the cells were collected and washed with PBS, and cell lysates were prepared in either RNAzol for RNA extraction or Radioimmunoprecipitation assay (RIPA) buffer for Western blotting.

RT-PCR using 1 µg RNA was conducted with the following primer pairs: F2: 5′-GACCACCTAGTTAGTCAAGG-3′ (end of Pol) and R1: 5′-CTTCTGGGTACTACCTTAATGTC-3′ (in Tat) using the Superscript III One-step RT-PCR system with platinum Taq DNA polymerase (Invitrogen; #12574-018). The thermocycler cDNA synthesis and pre-denaturation program was 45°C for 30 min; 94°C for 2 min; 40 cycles of denaturation at 94°C for 15 sec, annealing at 58°C for 30 sec, and extension at 68°C for 60 sec; and final extension at 68°C for 5 min.

Cell lysates were prepared in Radioimmunoprecipitation assay (RIPA) buffer, denatured at 95°C for 5 min, separated through polyacrylamide gel electrophoresis (PAGE), and transferred to polyvinylidene difluoride (PVDF) membranes. Afterward, they were washed with Tris-buffered saline (TBS) tween for 5 min and blocked for 1 hr at room temperature in TBS plus 5% milk. Next, 1:1,000 dilutions of the primary rabbit antibodies were used (Abeomics, San Diego, CA, USA; activated caspase 8 #20-1043, BAX #20-1022, and BAK #20-1019) along with mouse antibody to the control GAPDH (1:2,000) protein (Invitrogen; #MA5-15738) and incubated overnight. The next day, the membranes were washed thrice with the Tris-buffered saline tween (TBST) and incubated with 1:5,000 anti-rabbit AP and anti-mouse AP secondary antibody for 1 hr. The membranes were washed again thrice with TBST and developed using Promega (Madison, WI, USA) Western Blue Stabilized Substrate for Alkaline Phosphate #S3841.

#### 2.2.4 Annexin V and live/dead staining

In a 6-well plate, 5 × 10^5^ adherent HEK293 cells were transiently transfected using 9 µg PEI and 3 µg DNA (CSH-DIN-T/F Z331-BAX, CSH-DIN-T/F Z331-Cas8, or CSH-DIN-T/F Z331). The transfection medium was removed, and fresh medium was added after 8 hrs. Seventy-two hours post-transfection, 1 million cells were washed twice, stained with the Alexa Fluor 430 NHS Ester to exclude dead cells, and incubated for 15 min at room temperature. The cells were washed and stained in 25 mM HEPES buffer (100 µL) with Annexin V-FITC (5 µL) (BioLegend, San Diego, CA, USA; #640905), incubated at room temperature for 15 min, and washed twice, and a minimum of 10,000 events were acquired on a BD FACSAria Fusion (BD Biosciences, San Jose, CA, USA) instrument with the FACSDiva software package, and the data were analyzed using FlowJo Version 10.7.1 (Treestar, Woodburn, OR, USA).

### 2.3 Evaluation of safety, toxicity, and immunogenicity in mice

#### 2.3.1 Ethical statement regarding animal studies

Mice were housed in compliance with the “Guide”, the Animal Welfare Act (AWA), and Animal Welfare Regulations (AWR). All studies were approved by the University of Louisiana at Lafayette IACUC (2019-8802-030). Five mice were housed per cage to allow social interactions, with enrichment such as cellulose, pieces of wood, or cardboard rolls. The housing of mice was under controlled conditions of humidity, temperature, and light (12-hr white light/12-hr red light). Water and dry pellets were provided. Mice were anesthetized with isoflurane vapor (Patterson Veterinary) and monitored until awake. A group of 4–6-week-old BALB/cJ male/female mice were purchased from (Jackson Laboratory, Bay Harbor, ME, USA). They were housed in the New Iberia Research Center (NIRC).

To test for the absence of the integration of the vaccines, BALB/cJ mice were immunized i.m. and i.d. + electroporation with the CSH-DIN-T/F CAS8 DNA vaccine. Two weeks post-immunization, the mice were sacrificed, and DNA was extracted from the skin and muscle areas corresponding to the immunization sites and from the tail, spleen, and blood. PCR was conducted to amplify the end of Pol–beginning of Tat region with the primers F1 (5′-GCACACAAAGGTATAGGAGGAA-3′)—Primer R2 (5′-CTTCTGGGTACTACCTTAATGTC-3′). Mouse GAPDH housekeeping gene used as a sample loading control mus GAPDH Fwd 5′-AGGTCGGTGTGAACGGATTT-3′ mus GAPDH Rev2-5′-TGTAGACCATGTAGTTGAGGTCA-3′.

Skin and muscle tissues corresponding to the immunization sites were also fixed, embedded, and stained with hematoxylin and eosin (H&E) to evaluate for potential toxicity/inflammation 2 weeks post-immunization. Tissues were fixed in 4% PFA and embedded in paraffin (formalin-fixed, paraffin-embedded (FFPE) blocks). H&E slides from FFPE tissue blocks were analyzed, and the images were acquired on an Olympus IX71 microscope using the cellSens software.

The mice were distributed in groups. The mice were immunized with i.m. 50 µg DNA and i.d. 50 µg DNA with electroporation as described by Leroy et al. (Leroy et al., 2022). Different prime and boost approaches were tested with CSH-DIN-T/F Z331-BAX, CSH-DIN-T/F Z331-Cas8, or CSH-DIN-T/F Z331 DNA. At necropsy, the spleen was collected to isolate splenocytes for evaluation of the cell-mediated immune responses, while the blood was collected by intra-cardiac puncture to collect the serum for evaluation of the humoral immune responses.

#### 2.3.2 SHIV-specific intracellular cytokine determination

Mouse splenocytes were isolated and used to evaluate SHIV C-specific T-cell immune responses by multiparametric flow cytometry as previously described (Leroy et al., 2022). Briefly, 1 million cells were stimulated for 6 hrs with 2 µg/mL of each peptide pool (overlapping 15-mer with 11-aa overlaps), spanning the entire SIV Gag, HIV clade C Env [Env N terminal pool (1–435), HIV Env C terminal pool (436–857), or HIV clade C–Nef (AIDS Reagent Repository, NIH-NIAID catalog numbers 6204, 9499, and 13091, respectively)], or with phorbol myristate acetate (PMA)/ionomycin as positive control to assess the phenotype and cytokine production of vaccine-specific T cells. Non-stimulated cells were used to subtract the basal secretion signal. Splenocytes were stimulated for 6 hrs at 37°C. Anti-CD28 and CD49d antibodies (0.5 µg/mL) were added for co-stimulation, and Brefeldin A was added to prevent cytokine excretion (Leroy et al., 2022). To avoid the CD62L cleavage, 35 µg/mL of TAPI-2 (Sigma, Burlington, MA, USA) was added after 1-hr incubation with peptide pools. Splenocytes were then stained with surface and intracellular antibodies as described by Leroy et al. (Leroy et al., 2022). Antibodies were purchased from BD and eBioscience (San Diego, CA, USA) (**Table 1**). A minimum of 200,000 events were acquired on a BD FACSAria Fusion instrument with the FACSDiva software package, and the data were analyzed using FlowJo Version 10.7.1 (Treestar, USA).

**Table 1:** Panel of anti-mouse antibodies used for ICS assay.

Antibody	Clone	Conjugation	Company	Cat. no.
CD3	17A2	Alexa fluor 700	BD	561388
CD4	GK1.5	APC-H7	BD	560181
CD8a	53-6.7	BV605	BD	563152
CD127	SB/199	PE-Cy7	BD	560733
CD62L	MEL-14	PE-Cf594	BD	562404
IL-21	FFA21	eFluor 450	Invitrogen	48-7211-82
IL-2	JE56-5H4	APC	Invitrogen	17-7021-82
IFN-y	XMG1.2	BV711	BD	564336
CD16/CD32	2.4G2	Purified NA/LE	BD	553140
TAPI-2		1 mg	Sigma	SML0420
Brefeldin A			BioLegend	420601

#### 2.3.3 Systemic HIV clade C Env-ELISA-specific IgG binding antibody responses

Plasma binding antibodies to HIV-1 clade C Env protein were assessed by ELISA as described previously (Bose et al., 2024). Briefly, 96 wells (Nunc MaxiSorp™ flat bottom, Thermo Fisher Scientific, Waltham, MA, USA) were coated overnight at 4°C with HIV-1/Clade C gp120 recombinant protein (Acro, Newark, DE, USA; #16055) 2 μg/mL in bicarbonate buffer pH 9.6. It was then washed and blocked with 2% casein in PBS. The mouse serum was 1:4 diluted in casein and incubated for 1 hr at 37°C. Horseradish peroxidase (HRP)-conjugated goat anti-mouse IgG (1:20,000 dilution; Invitrogen; #31430) and the TMB substrate (KPL) were used in sequential steps, followed by reading the absorbance at 450 nm using a BioTek Synergy HT microplate reader.

### 2.4 Statistics

Statistical analysis was determined using the GraphPad Prism 8 software (Version 8.4.3). Medians were used for the different graphic presentations. Non-parametric Mann–Whitney, parametric one-way ANOVA, two-way ANOVA, and Tukey’s multiple comparison tests were used to determine the significance.

## 3 Results

### 3.1 *In vitro* characterization of CSH-DIN-T/F DNA vaccines with apoptotic genes

#### 3.1.1 HEK293 transfections and CEMx174 infections

All three prototype vaccines were tested *in vitro* to characterize them. The DNA vaccine constructs CSH-DIN-T/F Z331-BAX, CSH-DIN-T/F Z331-BAK, CSH-DIN-T/F Z331-Cas8, and CSH-DIN-T/F Z331 were transfected into HEK293 and co-cultured with CEMx174. As seen in **Figure 1B**, the SIV Gag p27 production peaked by day 5 post-transfection, with the highest concentration for CSH-DIN-T/F Z331-Cas8 followed by CSH-DIN-T/F Z331-BAX and the parent CSH-DIN-T/F Z331, while CSH-DIN-T/F Z331-BAK did not produce any measurable SIV Gag p27. Overall, these levels were lower than those obtained with a more active promoter such as the Cytomegalovirus Virus (CMV) one (Liu et al., 2007; Ganguly et al., 2010). Next, a passage of 100-µL day 2-infected HEK293 supernatants to CEMx174 cells showed only decreased input virus but no true reinfection, confirming the single-cycle nature of these DNA vaccines that are unable to integrate (**Figure 1C**).

#### 3.1.2 TZM-bl transfections and infections

Next, the number of cells transfected by CSH-DIN-T/F Z331-BAX, CSH-DIN-T/F Z331-BAK, CSH-DIN-T/F Z331-Cas8, and CSH-DIN-T/F Z331 were compared by exposure to TZM-bl cells. One hundred microliters of (4.8 × 10^8^ TCID_50_) the infectious replication-competent virus SHIV-C109p5 (Bose et al., 2024) was run in parallel as a positive control. At 72 hrs post-transfection, a total of 276 infected blue cells were counted for replication-competent virus SHIV-C109p5, while the blue-stained cells for CSH-DIN-T/F Z331-BAX and CSH-DIN-T/F Z331-Cas8 were 80 and 60, respectively. In contrast, CSH-DIN-T/F Z331-BAK and CSH-DIN-T/F Z331-Z331 had only four blue cells each (**Figure 1D**), reflecting the amount of p27 for the various constructs (**Figure 1B**).

#### 3.1.3 Detection of apoptotic gene products and function

##### 3.1.3.1 RT-PCR and Western blotting

**Figure 2:**
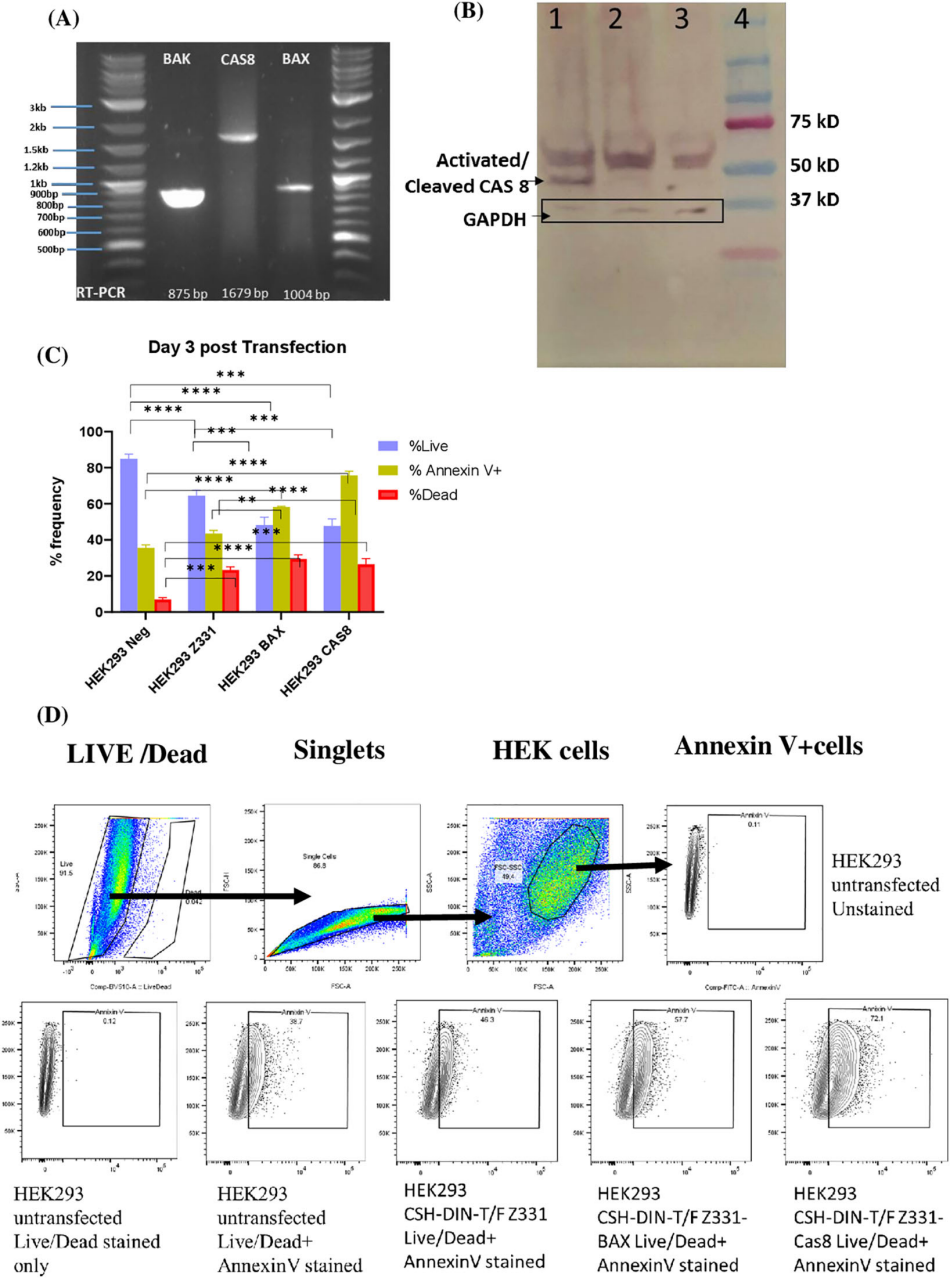
**(A)** Forty-eight hours post-transfection, HEK293 cells RNA was extracted to perform RT-PCR (1 µg RNA) using primer pairs complementary to the end of *pol* and the beginning of *tat*. The expected product sizes of the inserted apoptotic genes BAK, Cas8, and BAX were 875, 1,679, and 1,004 bp, respectively. **(B)** Western blotting was conducted with lysates from HEK293 cells transfected with the vaccine constructs CSH-DIN-T/F Z331-Cas8, CSH-DIN-T/F Z331, and un-transfected cells. Western blotting with the 48 hrs post-transfection cell lysates. Primary antibody: Anti-CAS8 Rabbit IgG Abeomics #20-1043(1:1,000) + Anti-GAPDH Mouse IgG Invitrogen #MA5-15738 (1:2,000). Secondary antibody: Goat anti-Rabbit AP + Goat anti-mouse AP (1:5,000). Lane 1: HEK293T Cell lysates from 48-hr post-transfected CSH-DIN-T/F Z331-Cas8 DNA. Lane 2: HEK293T Cell lysates from 48-hr post-transfected CSH-DIN-T/F Z331 DNA. Lane 3: Un-transfected HEK293T cell lysates. Lane 4: Precision Plus Protein Dual Color Standards (Bio-Rad, Hercules, CA, USA). **(C)** Transiently transfected cells with the lenti vector DNA vaccines were stained with the live/dead marker and Annexin V at 72 hrs post-transfection, and data were acquired on FACSAria. The graph summarizes the percentage of live cells, dead cells, and Annexin V-positive cells based on gated HEK cells. Experiments were conducted in triplicates, and the results were compared using Tukey’s multiple comparison test and two-way ANOVA. Error bars represent standard errors of the mean (s.e.m). **p < 0.01, ***p < 0.001, ****p < 0.0001. **(D)** The cells were gated first to select the live singlet cells and then gated for Annexin V positive. The gating was based on the unstained controls.

The RT-PCR products amplified from RNA extracted from 48 hrs post-transfected HEK293 cells corresponded to the expected sizes of the BAK, Cas8, and BAX (875, 1,679, and 1,004 bp, respectively) gene inserts (**Figure 2A**).

Western blotting from HEK29 cell lysates collected 48 hrs post-transfection with CSH-DIN-T/F Z331-Cas8 and the parent CSH-DIN-T/F Z331 was analyzed as described (**Figure 2B**). While both lysates comprised the full-length 55-kDa caspase 8 protein, only the CSH-DIN-T/F Z331-Cas8 cell lysate exhibited the activated caspase (p43/p41) 46.5-kDa band, demonstrating the activation of the apoptotic pathway. Similar analyses of BAX and BAK did not show any measurable difference, and these were constitutively expressed and did not have a distinct activated form (data not shown).

##### 3.1.3.2 Annexin V apoptosis assay

Annexin V detection was performed on HEK293 transfected with CSH-DIN-T/F Z331-BAX, CSH-DIN-T/F Z331-Cas8, and CSH-DIN-T/F Z331 at 72 hrs post-transfection. Owing to its minimal p27 production *in vitro*, the CSH-DIN-T/F Z331-BAK vaccine prototype was dropped from further analyses. Percentages of Annexin V-positive apoptotic cells were higher at 72.1% in CSH-DIN-T/F Z331-Cas8 followed by 57.7% in CSH-DIN-T/F Z331-BAX and 46.3% in CSH-DIN-T/F Z331. The percentage of dead cells was 29.3%, 33%, and 25.9% in CSH-DIN-T/F Z331-Cas8, CSH-DIN-T/F Z331-BAX, and CSH-DIN-T/F Z331, respectively (**Figures 2C, D**), confirming the enhanced induction of apoptosis with the vaccine prototypes with apoptotic genes.

### 3.2 Vaccine safety and immune responses in BALB/cJ mice

BALB/cJ mice were immunized intramuscularly (50 µg) and intradermally (50 µg) + electroporation with the CSH-DIN-T/F CAS8 DNA vaccine. Two weeks post-immunization, the mice were sacrificed, and DNA was extracted from the immunization sites, with muscles collected from the immunized area along with the tail, spleen, and blood. There was no evidence of DNA vaccine persistence (i.e., integrated DNA) from in the sites of injection: tail, spleen, or blood (**Supplementary Figure S1**). Further, 2 weeks post-immunization, the vaccine was tested for evidence of inflammation. There was no evidence of leucocyte infiltration at either site of immunization with the CSH-DIN-T/F CAS8 DNA vaccine, suggesting an absence of toxicity (**Supplementary Figure S2**).

#### 3.2.1 CSH-DIN-T/F Z331-Cas8 vaccine induces enhanced Env and Nef-specific cytotoxic T-cell responses

**Figure 3:**
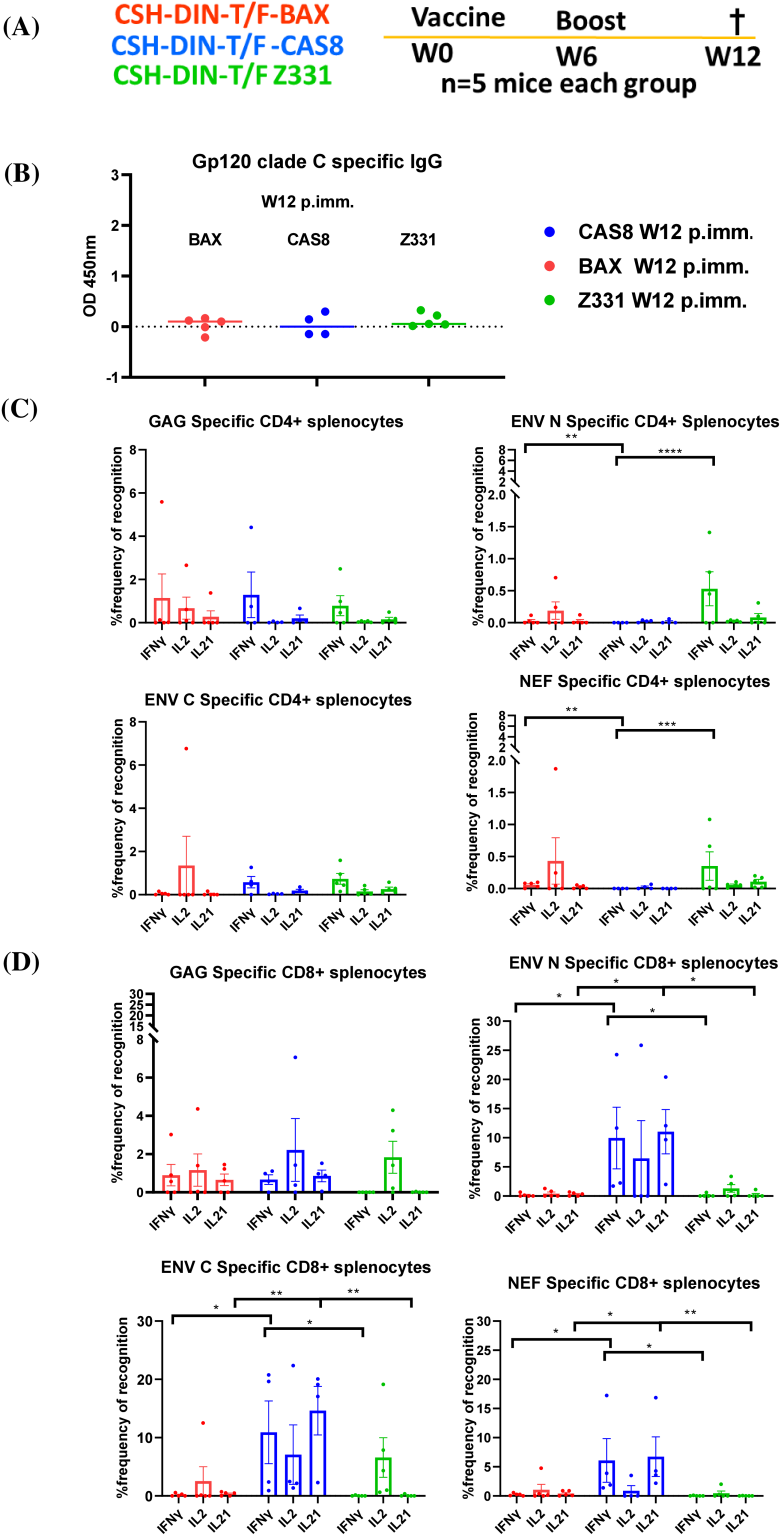
**(A)** Schematic of the *in vivo* studies on BALB/cJ. Lenti DNA vaccines were used to immunize mice i.m., and i.d. + electroporation at week 0, boosted at week 6, and mice were euthanized at week 12 post-immunization (W12 p. imm.); the serum and splenocytes were collected at necropsy. **(B)** gp120 clade C-specific IgG humoral responses of the different mouse groups were determined in the serum samples collected at week 12. **(C)** SHIV-specific IFN-γ-, IL-2-, and IL-21-producing CD4+ T cells. **(D)** CD8+ T cells from splenocytes of immunized BALB/cJ mice at week 12 post-immunization. Results were compared using paired t-test. Error bars represent standard error of mean (s.e.m), n = 5. *p < 0.05, **p < 0.01, ***p < 0.001, ****p < 0.0001, NS, not significant (p > 0.05). Statistical differences between total responses of each of the groups are indicated on the graph.

The first set of BALB/cJ mouse experiments were conducted by immunizing five mice each with CSH-DIN-T/F Z331-BAX, CSH-DIN-T/F Z331-Cas8, and CSH-DIN-T/F Z331. Due to the lack of p27 production by CSH-DIN-T/F Z331-BAK in *in vitro* studies, this vaccine prototype was not tested *in vivo*. Vaccines were delivered intramuscularly (50 µg) and intradermally (50 µg) with electroporation at week 0 and boosted at week 6. Mice were euthanized at week 12 post-vaccination (**Figure 3A**), and serum and splenocytes were collected at necropsy. The HIV clade C Env-specific humoral responses at the end of the experiment were barely above the background for all three vaccine prototypes tested (**Figure 3B**). Antigen-specific cellular responses were, however, readily detected in restimulated splenocytes. CD4+ and CD8+ T-cell responses were compared between vaccine prototypes for IL-2, IFN-γ, and IL-21 cytokine responses to Gag, Env N terminal (aa 1–435), HIV Env C terminal (aa 436–857), or HIV clade C–Nef peptide pools.

CSH-DIN-T/F Z331-BAX and CSH-DIN-T/F Z331-Cas8 induced higher CD4+ T cell Gag-specific IFN-γ responses compared to CSH-DIN-T/F Z331. There were also significantly higher CD4+ T cell IFN-γ immune responses specific to Env (N term) and Nef in cells from CSH-DIN-T/F Z331-BAX and CSH-DIN-T/F Z331 immunized mice compared to CSH-DIN-T/F Z331-Cas8 (**Figure 3C**).

The CSH-DIN-T/F Z331 parent vector induced IL-2 CD8+ T-cell responses to Gag and Env. CSH-DIN-T/F Z331-BAX induced polyclonal Gag-specific CD8+ responses but relatively few responses to Env or Nef. In contrast, CSH-DIN-T/F Z331-Cas8 induced the highest responses overall to all antigens (Gag, Env, and Nef), responding with IFN-γ, IL-2, and uniquely IL-21 (**Figure 3D**).

#### 3.2.2 CSH-DIN-T/F Z331-Cas8 induces memory late recall responses

**Figure 4:**
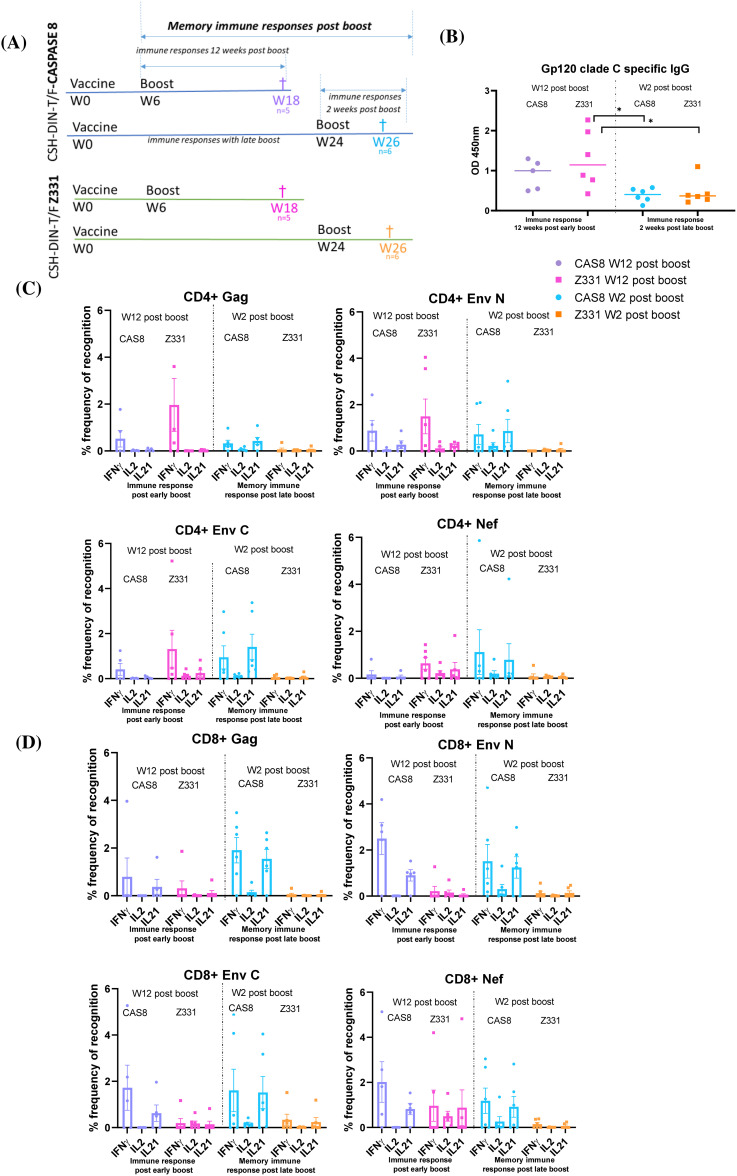
Schematics representing CSH-DIN-T/F Z331-Cas8 and CSH-DIN-T/F Z331 groups of BALB/cJ mice immunized to compare immune responses of early boost at week 6 (euthanized at week 12 post-boost) and late week 24 boost (euthanized week 2 post-boost). **(B)** gp120 clade C-specific IgG humoral responses of the different mouse groups were determined using the serum samples taken at week 18 post-immunization (week 12 post-boost) or week 26 post-immunization (week 2 post-boost). **(C)** SHIV-specific IFN-γ-, IL-2-, and IL-21-producing CD4+ T cells. **(D)** CD8+ T cells from splenocytes of immunized BALB/cJ mice at week 18 post-immunization (week 12 post-boost) or week 26 post-immunization (week 2 post-boost). Results were compared using paired t-test. Error bars represent the standard error of the mean (s.e.m), n = 5. *p < 0.05. Statistical differences between the total responses of each of the groups are indicated on the graph.

The ability to induce memory responses after early-boost (week 6 boost/necropsy collection at week 18; that is, 12 weeks post-boost) vs. late-boost (week 24/necropsy collection at week 26; that is, 2 weeks post-boost) recall responses were tested comparing CSH-DIN-T/F Z331-Cas8 with CSH-DIN-T/F Z331 (**Figure 4A**). Unexpectedly, no significant difference was observed in antibody titers to HIV clade C Env between the two vaccines CSH-DIN-T/F Z331-Cas8 and CSH-DIN-T/F Z331, indicating that the addition of the apoptotic genes did not influence the generation of humoral immune responses (**Figure 4B**). However, there was a marked difference in the kinetics of immune responses with the serum samples collected 12 weeks after the early boost given markedly higher titers than those collected 2 weeks after the late boost, perhaps due to a slower antigen expression or the decay of primed B cells. In these immunization schedules, late CD4+ T-cell responses to an early boost showed predominantly IFN-γ responses against all three antigens, although higher for CSH-DIN-T/F Z331 compared to CSH-DIN-T/F Z331-Cas8 (**Figure 4C**). Responses to the late boost, however, did not seem to generate higher recall responses with CSH-DIN-T/F Z331, while CSH-DIN-T/F Z331 Cas8 induced polyclonal responses to all three antigens, although perhaps the collections were not conducted at the peak responses. CD8 responses by contrast clearly reflected a far more potent immunization delivered by CSH-DIN-T/F Z331-Cas8 (**Figure 4D**). Not only did the late IFN-γ and IL-21 responses to an early boost persist for 3 months post-boost, but the late boost also recalled high responses for this construct, while responses to the parent CSH-DIN-T/F Z331 were very low overall.

#### 3.2.3 CSH-DIN-T/F Z331-Cas8 induces durable and late recall responses

**Figure 5:**
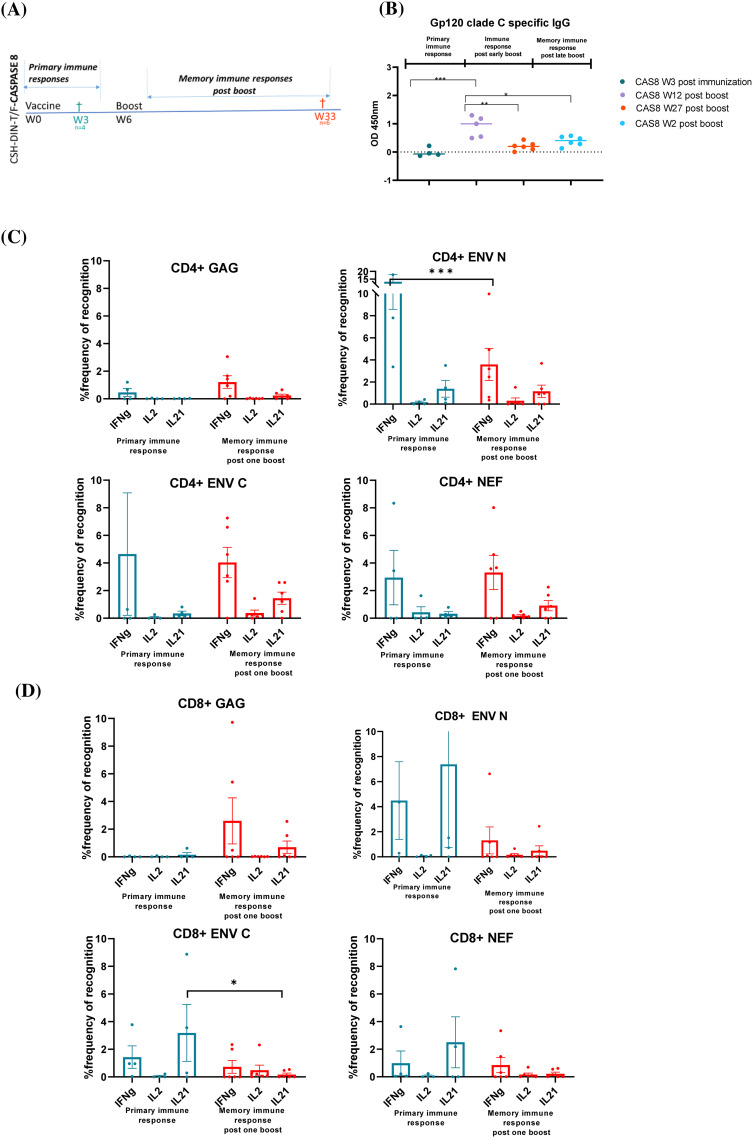
Schematics representing multiple groups of BALB/cJ mouse immunizations to compare the primary immune responses at week 3 post-immunization with memory immune responses at week 33 (week 27 post-boost). **(B)** gp120 clade C-specific IgG humoral responses of the different groups immunized with CSH-DIN-T/F Z331-Cas8 were compared. **(C)** SHIV-specific IFN-γ-, IL-2-, and IL-21-producing CD4+ T cells, **(D)** CD8+ T cells from splenocytes of immunized BALB/cJ mice at week 3 and week 33. Results were compared using the paired t-test. Error bars represent the standard error of mean (s.e.m), n = 5. *p < 0.05, **p < 0.01, ***p < 0.001. Statistical differences between total responses of each of the groups are indicated on the graph.

To further examine the longevity of the immune responses induced by CSH-DIN-T/F Z331-Cas8, immune responses observed 3 weeks after primary immunization were compared with memory responses 6 months after an early boost (**Figure 5A**). No antibody responses were detected 3 weeks after the prime immunization, but the highest HIV clade C Env antibody titers were observed with the CSH-DIN-T/F Z331-Cas8 vaccine 12 weeks post-first boost (**Figures 4B**, **5B**), suggesting the need for time to develop such antibodies after this type of immunization (**Figure 5B**). Memory humoral responses also showed marked decay since the mice collected at week 33, 6 months after an early boost, had markedly lower titers. Somewhat surprisingly, CD4+ IFN-γ responses to Env and Nef were high 3 weeks after the prime immunization, although with marked individual differences between animals from the same group (**Figure 5C**). Similarly, the CD8+ Env N or C term-stimulated splenocytes had higher IFN-γ and IL-21 responses at 3 weeks post-prime immunization, compared to memory responses post-boost (**Figure 5D**). In contrast, both CD4+ and especially CD8+ Gag responses were undetectable after the prime immunization while showing strong responses 6 months after the boost.

These data confirm that the addition of an apoptotic gene to the vaccine enhances cytotoxic T cell-specific responses.

## 4 Discussion

A safe and effective vaccine that prevents the acquisition or controls the replication of HIV remains elusive so far. While strategies to induce broadly neutralizing antibodies are progressing (Haynes et al., 2023; Nkolola and Barouch, 2024), these approaches would likely have minimal benefit past HIV acquisition. Hence, developing parallel strategies designed to enhance T-cell responses remains critical. To that extent, DNA vaccines have shown promise in generating cell-mediated immune responses especially when combined with electroporation to enhance transduction and antigen expression *in vivo* (Arrode et al., 2007; Arrode-Bruses et al., 2014; Chebloune et al., 2020). Our study was built on the premise that a vaccine expressing most lentiviral antigens may have one of the best abilities to induce broad and protective immune responses to HIV, even if the approach was clearly not able to prevent virus acquisition. The substitution of HIV/SIV LTR for the constitutively active CAEV LTRs has been shown to induce detectable immune responses in mice and monkeys as well as induce a rapid control of SIV infection (Arrode et al., 2007; Arrode-Bruses et al., 2014; Chebloune et al., 2020). This correlated with the presence of antigen-specific highly recallable T cells from precursors named PHPC (Arrode-Bruses et al., 2014). However, the vaccine had ample room for improvement in its induction of antiviral responses. In an initial approach, the inclusion of IL-7 and IL-15 as natural adjuvants was attempted, showing some modest benefits in mice and rhesus macaques (Leroy et al., 2022). In the current approach, the vaccine was updated via the inclusion of a clade C T/F HIV envelope more representative of widely transmitted isolates in the developing world (Deymier et al., 2014). Next, we wanted to combine this approach with the enhanced induction of apoptosis to promote cross-presentation of antigen *via* MHC Class I (Huang et al., 2007). This approach has not previously been attempted with a lentiviral-based DNA vaccine construct. Three pro-apoptotic genes were selected, two targeting the intrinsic apoptotic pathway (BAX and BAK) and the third one focusing on the extrinsic pathway (caspase 8). The apoptotic cassettes were individually placed after the *pol* coding sequence to avoid overexpression and premature expression relative to antigen production (Bergmann-Leitner and Leitner, 2004; Kim et al., 2004). Surprisingly, CSH-DIN-T/F Z331-BAK was non-functional, failing to express either antigens or the BAK apoptotic gene (**Figure 1**) in spite of mRNA presence and fully verified sequence. The reason for such failure remains unclear at present but led us to drop this construct from further evaluation.

The two other vaccine prototypes considered for *in vivo* studies with BALB/cJ were CSH-DIN-T/F Z331-BAX and CSH-DIN-T/F Z331-Cas8, which appeared to produce more SHIV antigens than the parent CSH-DIN-T/F Z331 devoid of apoptotic gene (**Figures 1B, D**) *in vitro*, suggesting gain of fitness, at least in cell culture, especially for CSH-DIN-T/F Z331-Cas8. Moreover, CSH-DIN-T/F Z331-Cas8 was shown to not only express caspase 8 but also generate activated caspase 8 (**Figure 2B**), leading to the enhanced apoptosis of transduced cells (**Figures 2C, D**). The superiority of CSH-DIN-T/F Z331-Cas8 was also apparent *in vivo*, whereby mice immunized with this construct generated markedly higher antigen-specific CD8-mediated responses than mice immunized with CSH-DIN-T/F Z331-BAX or parent CSH-DIN-T/F Z331. Our findings confirm studies by Sasaki et al. in which DNA vaccines can be made more effective by co-delivering antigens of interest with pro-apoptotic genes. In their study, DNA vaccines co-expressing influenza virus hemagglutinin or nucleoprotein genes and mutant caspase genes have markedly increased the T-cell responses (Sasaki et al., 2001). The projected mechanisms targeted were the induction of apoptosis of the initial antigen-producing cells to generate apoptotic bodies that are engulfed by Langerhans cells in the skin and dendritic cells in the draining lymph nodes, leading to higher activation, and cross-presentation of antigen complexed to MHC class I to T cells. One limitation of our study is the lack of demonstration of such apoptosis and cross-presentation *in vivo*.

However, earlier studies in cancer immunology have adopted the opposite approach, comparing pro-apoptotic and anti-apoptotic genes in DNA vaccines (Kim et al., 2004) based on the assumption that prolonging the life of short-lived dendritic cells (DCs) will enhance antigen presentation, leading to better immune responses (Kim et al., 2003b; Kim et al., 2003a; Huang et al., 2007; Kang et al., 2007; Cheng et al., 2008). However, a subsequent study compared the inclusion of the apoptotic gene *Bax* with the anti-apoptotic *Bcl-X_L_*, co-injected i.d. with DNA expressing the glycoprotein B (gB) of herpes simplex virus (HSV)-1. In this study, mice immunized with pBax and pgB induced higher titers of antibody along with stronger lymphocyte proliferative responses and cytotoxic activity compared to those mice that received pgB alone or with pBcl-xl (Parsania et al., 2010). These data reinforced the idea that cells transfected with plasmid DNA encoding apoptotic genes result in the formation of apoptotic bodies, which are phagocytosed by the dendritic cells resulting in immune responses to the antigen (Kinsey et al., 2004). In addition, having the apoptotic gene incorporated in the same DNA expressing the antigens further ascertains that the pro-apoptotic effect will be exerted on the cells transfected with the antigen-producing construct as opposed to the injection of two separate DNA plasmids (Kinsey et al., 2004).

The multiple immunization studies showed not only the early generation of CD8 T cell-mediated immune responses as early as 3 weeks post-prime but also polyclonal responses, attesting to the quality of such responses. Moreover, these responses when boosted were shown to be highly durable, with extensive levels of CD8 T cells producing IFN-γ in response to SIV Gag restimulation even 6 months after the last boost and beyond. While other HIV immunization attempts in mice using plasmid DNA with or without molecular adjuvants gave similar magnitudes of cell-mediated responses (Liu et al., 2007; Ganguly et al., 2010; Kulkarni et al., 2013; Zhou et al., 2013; Xu et al., 2020), our approach induces durable responses that are not restricted to select HIV antigens but incorporate all viral proteins except for the integrase. Even if responses to some of these antigens are below detection or not tested here, priming will have occurred with considerable breadth breath of the responses expected upon infection post-challenge.

In conclusion, we submit that we have generated a safe and promising single-cycle lentiviral DNA vaccine capable of inducing potent and durable CD8 T cell antiviral responses important for protection even post-viral acquisition to control viral loads (Turk et al., 2013). Moreover, our DNA vaccine may be combined with other vaccines promoting the induction of humoral immune response for future testing in a humanized mouse or non-human primate model of HIV infection.

## Data Availability

The raw data supporting the conclusions of this article will be made available by the authors, without undue reservation.
