# Supplementary figures and images for: Enhanced HIV immune responses elicited by an apoptotic single-cycle SHIV lentivector DNA vaccine
Source: Front Cell Infect Microbiol. 2025 Apr 10;15:1481427. doi: 10.3389/fcimb.2025.1481427 (PMC12023480; doi:10.3389/fcimb.2025.1481427)

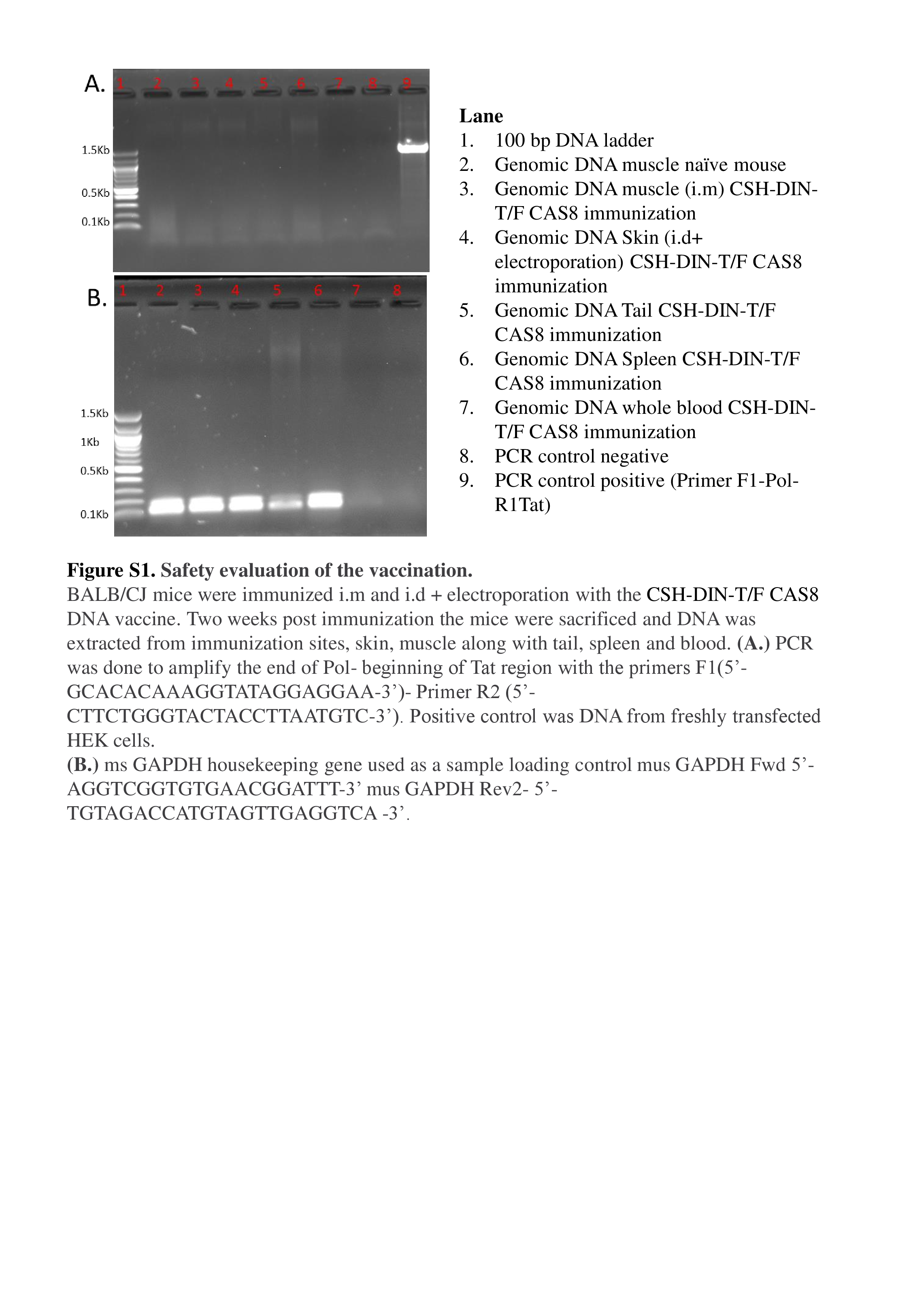

Supplement: Supplementary file 1 [file Image1.tiff]

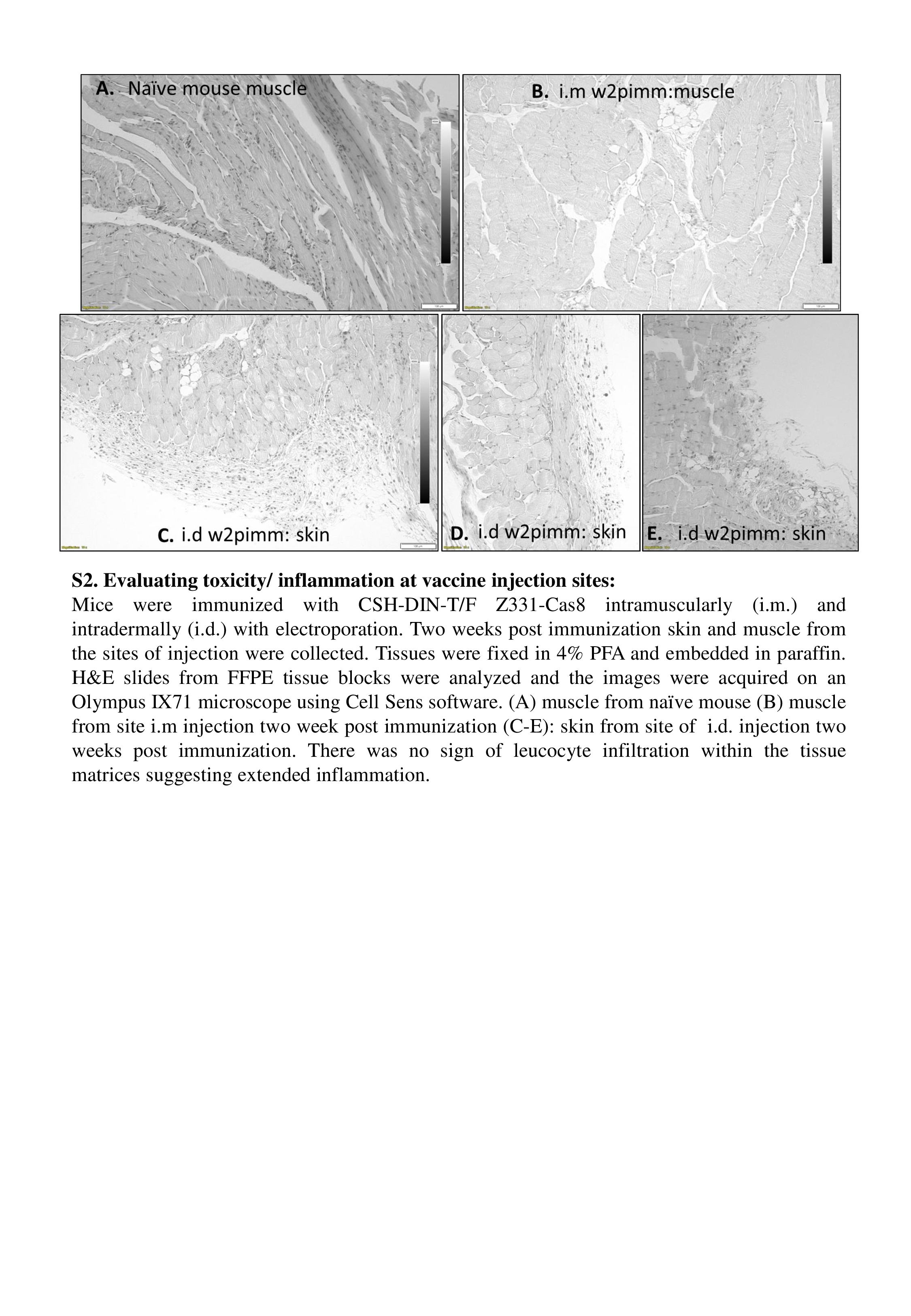

Supplement: Supplementary file 2 [file Image2.tiff]
